# Differences in tidal breathing between infants with chronic lung diseases and healthy controls

**DOI:** 10.1186/1471-2431-5-36

**Published:** 2005-09-08

**Authors:** G Schmalisch, S Wilitzki, RR Wauer

**Affiliations:** 1 Clinic of Neonatology (Charité), Humboldt-University of Berlin, Germany

## Abstract

**Background:**

The diagnostic value of tidal breathing (TB) measurements in infants is controversially discussed. The aim of this study was to investigate to what extent the breathing pattern of sleeping infants with chronic lung diseases (CLD) differ from healthy controls with the same postconceptional age and to assess the predictive value of TB parameters.

**Methods:**

In the age of 36–42 postconceptional weeks TB measurements were performed in 48 healthy newborns (median age and weight 7d, 3100 g) and 48 infants with CLD (80d, 2465 g)) using the deadspace-free flow-through technique. Once the infants had adapted to the mask and were sleeping quietly and breathing regularly, 20–60 breathing cycles were evaluated. Beside the shape of the tidal breathing flow-volume loop (TBFVL) 18 TB parameters were analyzed using ANOVA with Bonferroni correction. Receiver-operator characteristic (ROC) curves were calculated to investigate the discriminative ability of TB parameters.

**Results:**

The incidence of concave expiratory limbs in CLD infants was 31% and significantly higher compared to controls (2%) (p < 0.001). Significant differences between CLD infants and controls were found in 11/18 TB parameters. The largest differences were seen in the mean (SD) inspiratory time 0.45(0.11)s vs. 0.65(0.14)s (p < 0.0001) and respiratory rate (RR) 55.4(14.2)/min vs. 39.2(8.6)/min (p < 0.0001) without statistically significant difference in the discriminative power between both time parameters. Most flow parameters were strongly correlated with RR so that there is no additional diagnostic value. No significant differences were found in the tidal volume and commonly used TB parameters describing the expiratory flow profile.

**Conclusion:**

The breathing pattern of CLD infants differs significantly from that of healthy controls. Concave TBFVL and an increased RR measured during quiet sleep and under standardized conditions may indicate diminished respiratory functions in CLD infants whereas most of the commonly used TB parameters are poorly predictive.

## Background

With increasing numbers of infants born preterm, respiratory diseases associated with immature lungs and the need for mechanical ventilation or supplemental oxygen is becoming very common [1]. However, in the post surfactant era the "classic" or severe form of chronic lung disease (CLD) has been replaced by less severe forms ("mild" CLD) which are observed in very small premature infants who survive after prolonged mechanical ventilation [2,3]. Increasing awareness that both inflammation and disturbed lung development may cause respiratory problems in the middle and old age has emphasized the need for simple methods to assess lung function during the early age [4].

Several studies have shown that the degree of impaired respiratory function can be assessed by respiratory function testing [5-7]. However these complex techniques are limited to specialized centers. In contrast, tidal breathing (TB) measurements can be performed relatively easily in healthy and sick neonates at the bedside [8], and therefore they are frequently used for clinical and research purposes [9-11].

Air flow V'(t) and volume V(t) determined by numerical integration of V'(t) are the basic signals of a TB analysis. Both signals plotted together in an x-y diagram represent the tidal breathing flow-volume loop (TBFVL). In addition to assessing the shape of the TBFVL, several parameters were measured or derived from the time signals or the TBFVL to describe the breathing pattern.

Tidal breathing is commonly measured at the airway opening using a pneumotachograph (PNT) connected to a face mask [8]. In preterm and sick neonates, the total apparatus deadspace (V_D, app_) may exceed the infant's own deadspace and limits the time of measurement, even when a small face mask and a very low deadspace flow meter are used. Therefore for TB measurements in small infants, the deadspace-free flow-through technique (FTT) was developed [12] which virtually eliminates V_D, app _by a background flow. In contrast to other techniques, the elimination of V_D, app _by the FTT enables pneumotachographic long-term measurements which are an essential prerequisite for reliable TB measurements in infancy [12].

In the past tidal breathing measurements in CLD infants were commonly performed in infants with the severe form of CLD using the conventional technique (face mask and PNT) [13-15]. In these infants a significantly higher respiratory rate, a longer expiratory time, an earlier peak expiratory flow and concave expiratory limbs of the TBFVL were seen. Can we see such alterations also in the current milder forms of CLD and how many infants show such breathing pattern? Furthermore, Emralino and Steele [16] have shown that TB parameters measured with the conventional technique are significantly affected by the measuring technique itself and they cannot be compared with parameters measured during quiet breathing. Therefore the aim of this study was to investigate to what extent the breathing pattern of sleeping infants with the current mild forms of CLD differ from healthy controls with the same postconceptional age using the deadspace-free FTT and to assess the predictive value of the different TB parameters.

## Methods

### Subjects

In a prospective clinical study over 3 years TB measurements were performed in 96 infants (37 female and 59 male) with a postconceptional age between 36 and 42 weeks. All measurements were performed in the respiratory function laboratory of the Clinic of Neonatology at the Humboldt University (Charité). Inclusion criteria for this study were: spontaneous ventilation, quiet sleep according to Prechtl [17], and written parental consent.

Exclusion criteria were: upper respiratory airway disease and acute upper respiratory airway infection, congenital heart disease with exception of persistent ductus arteriosus, congenital diaphragmatic hernia and other pulmonary malformations, central nervous respiratory dysregulation.

In this study all CLD infants of the study period and the same number of healthy controls matched for postconceptional age were enrolled.

Group 1: 48 healthy newborns born in the Charité Hospital without any signs of respiratory disease or need for extra oxygen were recruited as controls.

Group 2: 48 infants with CLD came from different departments of neonatology. In our laboratory we measured our patients as well as patients admitted from other hospitals for pulmonary follow-up. All infants had a history of a severe postnatal RDS and required mechanical ventilation for >72 hours. The diagnosis of CLD was given by oxygen requirement at day 28 of age. Oxygen dependency was defined as the inability to keep oxygen saturation >92% in room air for at least 12 hours a day. 45/48 CLD infants were of a gestational age <32 weeks. 46/48 CLD infants were breathing room air at 36 weeks post-conception. According to the recently proposed diagnostic criteria of CLD by Jobe and Bancalari [3] the most CLD infants of this study had a mild form of CLD.

The patient characteristics are shown in Table 1. Although the controls were of the same postconceptional age, the controls were more mature at birth and heavier at the time of measurement. Because it is rare for infants born at 24 to 32 weeks gestational age not to develop any form of lung disease [5] a term control group of healthy newborns was used. Unfortunately, from the most of the outborn patients, no information was available about antenatal corticosteroids, postnatal surfactant treatment and the exact duration of mechanical ventilation and oxygen support, so that these important predictors could not be evaluated.

**Table 1:** Patient characteristics (median and range in brackets)

	**Healthy neonates **(n = 48)	**CLD infants **(n = 48)
Birth weight (g)	3280 (1610 – 4670)	890*** (450 – 3860)
Gestational age (weeks)	39 (34 – 41)	27*** (24 – 34)
Age (days)	7 (3 – 12)	86*** (33 – 125)
Postconceptional age (weeks)	39.6 (35.6 – 42.3)	38.7 (36.0 – 42.3)
Body weight at time of measurement (g)	3120 (1590 – 4580)	2400*** (1950 – 3800)

Comparison with healthy controls: *** p < 0.001

This study was approved by the ethical committee of the medical faculty (Charité) of the Humboldt University (protocol 54/92). Parents were given a full explanation of the tests and equipment used before their written consent was obtained.

### Equipment for TB measurements

Tidal breathing was measured as described previously [12] using custom made equipment based on the flow-through technique. Briefly, the face mask is continuously rinsed thoroughly by a constant background flow higher than the infant's peak tidal inspiratory flow. The flow in and out of a modified transparent face mask (Vital Signs Inc., Totowa, USA) were measured by two screen PNTs (Baby PNT Jaeger, Wuerzburg, Germany) with a low flow resistance (0.2kPa·L^-1^·s). The infant's tidal flow was measured by the difference between the two flow signals. Both PNTs were calibrated simultaneously with room air at the beginning of each measurements using a 100 mL calibration syringe (Hans Rudolph, Kansas City, USA). The continuous background flow, which was generated by an air mixer and flow regulator, did not have a significant effect on the calibration and measurement accuracy. For background flows up to 7L/min the in-vitro volume error was <3% [18]. Changes of temperature, humidity, and gas viscosity between calibration and measurement were numerically corrected by the software.

The flow signal was filtered by an analogue Bessel filter of 4^th ^order with 48Hz cutoff frequency to avoid aliasing, sampled with a 16 bit analogue/digital converter and recorded at 200 Hz.

### Protocol of lung function testing

All infants were tested when well and clinically free from an upper airway infection since ≥3weeks. Most infants were studied during natural, quiet sleep, assessed by behavioural criteria [17], but 12 infants (12%) (1 healthy neonate and 11 CLD infants) were sedated with chloral hydrate (50 mg·kg^-1^) given orally 15–30 minutes before testing.

Sleeping infants were in a supine position with the neck in a neutral position supported with a roll. The background flow was adjusted (about the six fold of the expected minute ventilation of 220 mL/kg [18]) before the face mask was placed. Only in few infants an increase of the background flow was necessary to prevent rebreathing. After a period of accommodation (5–20 min), TB was measured while the airtight seal of the mask on the infant's face was checked by continuous leak monitoring [19]. The end of the accommodation period is commonly characterized by a more regular breathing pattern without any visible drift in TB parameters [20]. The graphical display of the instantaneous respiratory rate over the last 60 breathing cycles was used to assess the stability of the TB parameters. The duration of the TB measurements was normally 20–30 minutes depending on the period of accommodation to the face mask. All infants were continuously monitored by pulse oxymetry to prevent any adverse event particularly in sedated infants [21]. Parents were usually present during the respiratory function testing.

**Figure 1:**
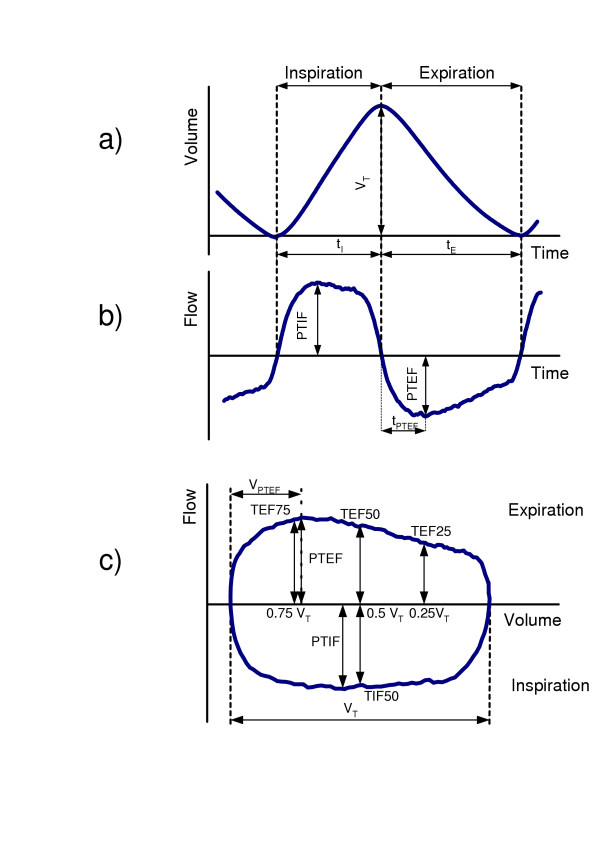
Tidal breathing parameters used in this study of a) the volume and b)the flow signals and c) the flow-volume loop. **Abbreviations**: t_I_-inspiratory time, t_E_-expiratory time, V_T_-tidal volume, PTIF, PTEF-peak tidal inspiratory and expiratory flow, t_PTEF_-time to peak tidal expiratory flow, TIF 50-tidal inspiratory flow when 50% of V_T _is inspired, V_PTEF _exhaled volume to peak tidal expiratory flow, TEF75, TEF50, TEF25- expiratory flow when 75%, 50% and 25% of tidal volume remains in the lung.

**Figure 2:**
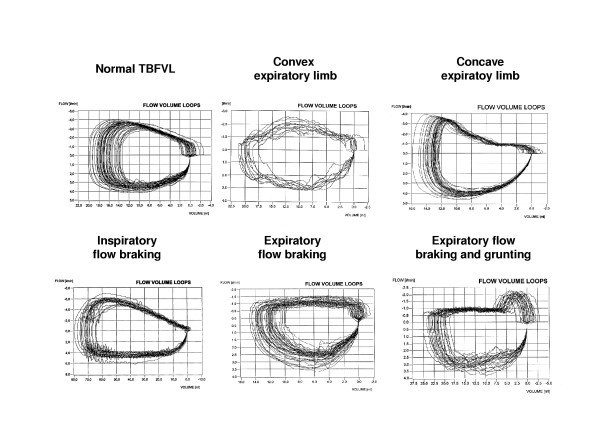
Typical shapes of tidal-breathing flow volume loops in newborns. To reduce the breath-to-breath variability a zeroing of volume at the begin of each inspiration was performed. In accordance with the common presentation of flow-volume loops, the inspiration started on the right side and continues in the lower quadrant, whereas the expiration follows in the upper quadrant.

Depending on the variability of the breathing pattern, an interval of 20–60 consecutive artifact-free breaths with the same basic pattern of the TBFVLs were selected by the investigator and evaluated by the software at bedside as shown in Fig. 1. A subjective influence of the investigator on the calculated TB parameters can be excluded. From the recorded breathing cycles an averaged breathing loop was calculated as described previously [22]. After finishing the study the averaged loops were blinded and classified by three investigators (GS, SW, RW) according to typical patterns shown in Fig 2. A majority vote was accepted in only a small number of cases where f the loop pattern could not be clearly identified by all three investigators.

For the quantitative evaluation, eleven basic parameters (Fig. 1) were measured from the flow and volume signals [inspiratory time (t_I_), expiratory time (t_E_), tidal volume (V_T_), tidal inspiratory flow when 50% of V_T _is inspired (TIF 50), peak tidal inspiratory and expiratory flow (PTIF, PTEF), time to peak tidal expiratory flow (t_PTEF_), exhaled volume to peak tidal expiratory flow (V_PTEF_) and expiratory flow when 75%, 50% and 25% of tidal volume remains in the lung (TEF75, TEF50, TEF25)]. From these parameters seven characteristic TB parameters were derived [respiratory rate (RR), minute ventilation (V'_E_), mean inspiratory flow V_T_/t_I_, mean initial expiratory gas acceleration PTEF/t_PTEF_, the ratios t_PTEF_/t_E_, V_PTEF_/V_T_, and the axis ratio of the TBFVL given by (PTIF+PTEF)/V_T_)].

### Statistical methods

Patient characteristics are recorded as the median and range and compared using the Wilcoxon, Mann Whitney test. Differences in the pattern of the TBFVL between the groups were tested by means of the Chi^2^-test. Mean and standard deviations (SD) were calculated for all TB parameters, and analysis of variance (ANOVA) with Bonferroni correction for multiple comparisons was used to quantify differences between the patient groups. Birth weigh and gestational age were taken as covariates to investigate the effect of prematurity on the difference in TB parameters. Receiver operating characteristic curves (ROC) were calculated to investigate the discriminative ability of tidal breathing parameters in order to distinguish breathing patterns of both patient groups. The 95% confidence interval of the area under the normalized ROC curve (AUC) was calculated as described by Hanley and McNeil [23]. A level of statistical significance of p < 0.05 was accepted.

## Results

The qualitative evaluation of the TBFVLs showed that the shape of the inspiratory limb was commonly convex and there were no statistically significant differences in the inspiratory shapes between the patient groups. In contrast to the inspiratory limb the shape of the expiratory limb after PTEF varied widely. Table 2 shows the distribution of typical patterns in both patient groups with significant differences (p < 0.001) in the distribution. As shown in this table, concave expiratory limbs were rarely seen in healthy infants (2%) but in about one third of all CLD infants.

**Table 2:** Distribution of typical shapes of the expiratory limb of the tidal breathing flow-volume loop after the peak tidal expiratory flow (PTEF) (absolute number and percentages in brackets)

Shape	**Healthy neonates **(n = 48)	**CLD infants **(n = 48)
Convex	10 (21%)	9 (25%)
Linear	31 (65%)	13 (27%)
**Concave**	**1 (2%)**	**15 (31%)**
Flow limitation	5 (10%)	5(11%)
Other shapes	1 (2%)	3 (6%)

The comparison of the TB parameters between CLD infants and healthy controls is shown in Table 3. Because the controls were significantly heavier at the day of measurement (Table 1), all flow and volume parameters were related to the body weight to reduce the inter-subject variability.

**Table 3:** Comparison of TB parameters between both patient groups ordered according to the p-value of the ANOVA (Presented are group means ± SD, statistically significant p-values after Bonferroni correction (p < 0.0028) are printed in bold)

**Parameter**	**Healthy neonates (n = 48)**	**CLD infants (n = 48)**	**p-value CLD**
t_I_(s)	0.65 ± 0.14	0.45 ± 0.11	**p < 0.0001**
RR (min^-1^)	39.2 ± 8.6	55.4 ± 14.2	**p < 0.0001**
(PTIF+PTEF)/V_T _(s^-1^)	0.27 ± 0.06	0.37 ± 0.09	**p < 0.0001**
t_E _(s)	0.98 ± 0.24	0.72 ± 0.22	**p < 0.0001**
V_T_/t_I_(mL·s^-1^·kg^-1^)	8.9 ± 2.2	11.6 ± 2.8	**p < 0.0001**
V'_E_(mL·min^-1^·kg^-1^)	215 ± 51.7	276 ± 76.8	**p < 0.0001**
TIF 50 (L·min^-1^·kg^-1^)	0.75 ± 0.21	0.98 ± 0.26	**p < 0.0001**
PTIF (L·min^-1^·kg^-1^)	0.83 ± 0.20	1.05 ± 0.28	**p < 0.0001**
PTEF/t_PTEF _(L·s^-2^·kg^-1^)	2.90 ± 1.68	5.25 ± 3.66	**p = 0.0001**
PTEF (L·min^-1^·kg^-1^)	0.64 ± 0.19	0.82 ± 0.29	**p = 0.0006**
t_ptef _(s)^*)^	0.24 ± 0.09	0.18 ± 0.11	**p = 0.001**
TEF75 (L·min^-1^·kg^-1^)	0.60 ± 0.20	0.76 ± 0.29	p = 0.003
TEF50 (L·min^-1^·kg^-1^)	0.52 ± 0.17	0.66 ± 0.25	p = 0.003
TEF25 (L·min^-1^·kg^-1^)	0.38 ± 0.11	0.46 ± 0.16	p = 0.006
V_ptef _(mL/kg)^*)^	1.68 ± 0.52	1.37 ± 0.44	p = 0.006
V_T _(mL·kg^-1^)	5.57 ± 1.06	5.15 ± 1.35	p = 0.09
V_PTEF_/V_T_(%)^*)^	29.4 ± 6.6%	27.2 ± 6.1%	p = 0.13
t_PTEF_/t_E _(%)^*)^	25.8 ± 9.7%	23.2 ± 7.8%	p = 0.20

^*)^Loops with flow limitations, grunting or other deformations were excluded from the evaluation (6 controls, 8 CLD infants)

**Abbreviations: **t_I,E_-inspiratory, expiratory time, RR-respiratory rate, PTIF, PTEF-peak tidal inspiratory and expiratory flow, V_T_-tidal volume, TIF 50-tidal inspiratory flow when 50% of V_T _is inspired, TEF 75, TEF 50, TEF 25-expiratory flow when 75%, 50% and 25% of tidal volume remains in the lung, V'_E_-minute ventilation, t_PTEF_,V_PTEF_-time and volume to peak tidal expiratory flow

The parameters in Table 3 were ordered according to the p-value of the ANOVA. The covariates birth weight and gestational age didn't have any statistically significant effect on the differences in TB parameters between the patient groups. The lowest p-values between the patient groups were found in the time parameters of the breathing cycle. The best discriminating parameter was t_I _whereas the differences in t_E _were distinctly lower. Nevertheless, the respiratory rate which is the reciprocal value of the sum t_I_+t_E _is one of the most important parameters distinguishing CLD infants from healthy controls.

The high differences in the axis ratio of the TBFVL (given by (PTIF+PTEF)/V_T_) between the groups can be explained by the strong correlation with RR. For a sinusoidal flow signal is RR = π·(PTIF+PTEF)/V_T_. Large differences were also found in the mean inspiratory flow V_T_/t_I_, minute ventilation V'_E_, and in the initial expiratory gas acceleration denoted as PTEF/t_PTEF_.

The differences between the patient groups in the flow parameters (PTIF, PTEF) are statistically significant, but considerably lower than the differences in RR. Furthermore, most flow parameters are strongly correlated with RR so that there is no additional diagnostic value. A surprising result is that the differences between the patient groups decreased in the course of expiration. In contrast to PTEF, the differences in the end-expiratory flow (TEF50, TEF25) are not statistically significant. No statistically significant differences were found in tidal volume related to the body weight and in the widely used TB-parameters t_PTEF_/t_E _and V_PTEF_/V_T_.

**Figure 3:**
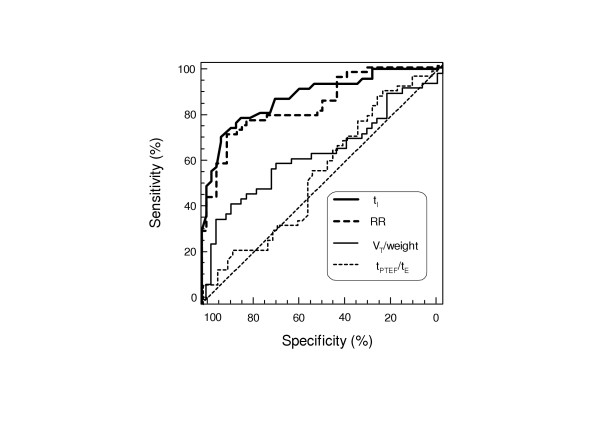
ROC curves of inspiratory time t_I_, respiratory rate (RR), tidal volume (V_T_) and the ratio t_PTEF_/t_E_between CLD infants and healthy controls.

The discriminative power of TB parameters between both patients groups was investigated by using the ROC analysis (Fig. 3). In Table 4 the area under the ROC curve (AUC), the optimal cut-off value for each significantly different TB parameter and the resulting sensitivity and specificity were presented. For the best discriminating parameters t_I _and RR the area under the curve was not statistically different. This means that there will not be a large difference in the diagnostic value of t_I _compared to the more commonly used parameter RR. The sensitivity of both parameters using the optimal cutoff value was the same (70.8%). The sensitivity of the other TB parameters was distinctly lower and likely too low for the most clinical applications.

**Table 4:** ROC analysis of commonly used TB parameters between CLD infants and healthy controls. If the 95% confidence interval (95% CI) of the area under the normalized ROC curve (AUC) include the 0.5 value (no discrimination) than there is no evidence that the TB parameters has the ability to distinguish between the two groups

**Parameter**	**AUC with 95%CI**	**Optimal cut-off point**	**Sensitivity**	**Specificity **
t_I_	0.879 (0.808 to 0.950)	0.48 s	70.8%	91.7%
RR	0.842 (0.754 to 0.909)	49.1 min^-1^	70.8%	89.6%
V_T_/t_I_	0.809 (0.721 to 0.896)	11.1 mL·s^-1^·kg^-1^	58.3%	89.6%
V'_E_	0.776 (0.682 to 0.869)	250 mL·min^-1^·kg^-1^	64.6%	85.4%
PTIF	0.747 (0.649 to 0.845)	0.95 L·min^-1^·kg^-1^	62.5%	79.2%
PTEF/t_PTEF_	0.688 (0.582 to 0.794)	4.57 L·s^-2^·kg^-1^	52.8%	87.5%
PTEF	0.673 (0.566 to 0.780)	0.79 L·min^-1^·kg^-1^	45.8%	83.3%
TEF25	0.653 (0.544 to 0.762)	0.67 L·min^-1^·kg^-1^	43.7%	87.5%
V_T_	0.610 (0.497 to 0.722)	-	-	-
V_PTEF_/V_T_	0.565 (0.450 – 0.620)	-	-	-
t_PTEF_/t_E _(%)	.552 (0.437 – 0.670)	-	-	-

(Abbreviation see Table 3)

## Discussion

The main goal of this study was to investigate to what extent the tidal breathing pattern of CLD infants differ from healthy controls. We found in CLD infants a high incidence (31%) of concave TBVFL and significant differences mainly in the time parameters of the breathing cycle. t_I _was decreased and RR was increased in about 71% of all CLD infants whereas the most TB parameters were poorly predictive

The fact that time parameters show the largest differences between CLD infants and controls indicate the main problem of TB measurements. It is well recognized by numerous studies that the respiratory rate of an infant is affected by the measurement equipment itself [12,16,24], the time of measurement [20], behavioural states [17] or non-pulmonary diseases (e.g. infections). Therefore, the standardization of equipment and measuring conditions is an urgently necessary to obtain reliable results.

### Techniques of tidal breathing measurements

For monitoring purposes tidal breathing in infants is commonly measured by indirect methods like breathing belts or measurement of transthoracic impedance changes. These techniques do not affect the air flow, however, accurate air flow measurements are only possible after circumstantial calibration and if measurements conditions are very stable [25]. Reliable TBFVL can not be obtained by indirect methods. Dead space free ventilatory measurements without any facial attachment are possible by "face out" body plethysmography. However, this technique is too expensive and cumbersome for routine bedside application [26]. Thus, the use of a face mask connected to a pneumotach is commonly used for precise ventilatory measurements [8].

The main problem of this conventional technique is the relatively high apparatus dead space which limits the duration of measurement due to CO_2 _rebreathing [27] so that a sufficient adaptation time after application of the face mask [28] can not be realized. Some of these influencing factors are eliminated by the FTT. The virtual elimination of the apparatus dead space by the background flow permits long-term measurements, so that the duration of measurements can readily be adapted to the prevailing measuring conditions (e.g., time required to reach a steady state) as well as to the variability of the respiratory signals. Furthermore, the airtight placement of the face mask can be monitored [19].

### TB-Parameters

In the present study, the differences in the TB parameters between the patient groups are in good agreement with published results. Hjalmarson and Sandberg [5] recently showed in a prospective clinical study that preterm infants with mild or moderate CLD had significant higher RR compared to controls but no statistically significant changes in V_T _related to body weight. Ranganathan et al. [29] found in young infants with cystic fibrosis an elevated RR but no significant changes in the commonly used TB parameters. Tepper et al. [14] reported a significantly higher RR in CLD infants but no significant changes in V_T_. In the present study the unchanged tidal volume related to body weight and the much higher RR in CLD infants compared to healthy neonates explain the significantly higher flow parameters. In contrast to tidal breathing, it is well recognized that at forced expiration CLD infants show a significant flow limitation due to poor growth of the airways and the resulting higher peripheral airway resistance [14]. During tidal breathing we have never seen a reduced end-expiratory flow (TEF25) in CLD infants probably due to their high RR and the resulting higher expiratory flow rates.

The parameters t_PTEF_, t_PTEF_/t_E _as well as V_PTEF_, V_PTEF_/V_T _(which are strongly correlated), describe the site of PTEF in the flow and in the TBFVL, respectively. These parameters were frequently used in the past to detect airway obstructions [10,30,31]. However, the association of these parameters with small airway caliber remain speculative and could not be demonstrated in previously published studies [32,33]. In the present study we found only for t_PTEF _a statistically significant reduction in CLD infants The higher PTEF and the shorter t_PTEF _in CLD infants explain the significant differences between the patient groups in the mean initial expiratory gas acceleration given by PTEF/t_PTEF_.

In a recent study we have shown [34] that TB parameters of newborns describing the flow profile or the shape of the TBFVL describe rather the breathing strategy than an impaired lung function. Neonates have a highly compliant chest wall which may cause several problems during breathing e.g., small end-expiratory lung volume, low oxygen stores, and a high risk for airway occlusion and atelectasis [35]. Therefore, infants compensate for this mechanical disadvantage by actively maintaining lung volume above the resting volume which affects significantly the measured TB parameters. This may explain the decreasing significant differences in the TB parameters between the patient groups in the course of expiration.

An unexpected finding of our recent study [34] was that conventionally used TB parameters (e.g., RR, V_T_, V'_E_, mean V'_I_) are relatively robust against changes in dynamically elevated lung volume. Furthermore, these parameters are clearly defined and easy to derive from the measured respiratory signals. Therefore, TB measurements in neonates should be focused much more on the evaluation of these conventional parameters measured under standardized conditions which had also shown in the present study the highest discriminative potential.

The interpretation of TB measurements remains difficult because they reflect both the control of breathing and respiratory mechanics. Thus the breathing pattern can be influenced by factors other than impaired respiratory mechanics (e.g. changes in glottic aperture) and a TB measurement can never reveal impaired respiratory functions with complete reliability, because there is always the chance that the breathing pattern has been affected by an abnormality in the neural control of breathing [36]. Nevertheless, concave expiratory limbs (Table 2) were nearly exclusively seen in CLD infants (31%) and in about 70% of the CLD infants, RR was notably increased. Thus, despite its methodological limitations TB measurements can give valuable information about impaired respiratory function which should be investigated more in detail by further methods.

There are several limitations to the interpretation of our results. First, the control group was more mature at birth than the CLD infants so that the difference in TB parameters could also be affected by the immaturity of the CLD infants. However, this could not be confirmed in the ANOVA using birth weight and gestational age as covariates. Second, with exception of one healthy infant sedation was only used in CLD infants (23%). However, we did not find any statistically significant differences in the TB parameters between sedated and unsedated infants which is in well agreement with earlier investigations [12]. Third, from the majority of the admitted CLD infants important predictors (e.g. administration of steroids, duration of mechanical ventilation and oxygen therapy) were not known so that their influence on the changes in tidal breathing could not be investigated.

### Reference ranges

The main problem of current TB measurements are the missing reference ranges. Despite repeated efforts during the last 50 years, the published reference values [11] are highly specific to the equipment used and the behavioral state of specific populations. They are unlikely to be of relevance when using other measurement techniques or equipment [12]. The fact that some TB parameters in neonates depend on the infant's breathing strategy makes it difficult to establish reference ranges for these parameters. In contrast, TB parameters describing breathing rate and depth are relatively independent from the breathing strategy and new efforts should be undertaken to determine reference ranges considering the biological development.

## Conclusion

Diminished respiratory functions in infants after neonatal intensive care may be derived easily and non-invasively by TB measurements. Beside the shape of the TBFVL, time parameters of the breathing cycle showed the highest sensitivity. However, reliable measurements are only possible during quiet sleep and under standardized long-term measurements. Although the breathing pattern is affected by both the neural control of breathing and respiratory mechanics, TB-measurements can be used as a first-line tool in the respiratory function testing of infants after neonatal intensive care. However, the causes of the diminished respiratory function have to be investigated by more specialized methods.

## Abbreviations

AUC – Area under the ROC curve

CLD – Chronic lung disease

FTT – Flow-through technique

PNT – Pneumotachograph

PTIF, PTEF – Peak tidal inspiratory and expiratory flow

RDS – Respiratory distress syndrome

ROC – Receiver operatic characteristic

RR – Respiratory rate

TB – Tidal breathing

TBFVL – Tidal breathing flow-volume loop

TEF 75, 50, 25 – Expiratory flow when 75%, 50% and 25% of V_T _remains in the lung,

t_I, E _– Inspiratory, expiratory time

TIF 50 – Tidal inspiratory flow when 50% of VT is inspired

tPTEF, VPTEF – Time and volume to peak tidal expiratory flow

*V*(t) – Volume

*V'*(t) – Air flow

V'_E _– Minute ventilation

*V*_D _– Dead space

V_T _– Tidal volume

## Competing interests

The author(s) declare that they have no competing interests.

## Authors' contributions

GS and RW had primary responsibility for study design, protocol development, data analysis and writing of the manuscript. SW carried out all lung function measurements and GS performed statistical analysis. All authors read and approved the final manuscript.

## Pre-publication history

The pre-publication history for this paper can be accessed here:


